# Prevalence and incidence of nodding syndrome and other forms of epilepsy in onchocerciasis-endemic areas in northern Uganda after the implementation of onchocerciasis control measures

**DOI:** 10.1186/s40249-020-0628-3

**Published:** 2020-03-02

**Authors:** Nolbert Gumisiriza, Frank Mubiru, Joseph Nelson Siewe Fodjo, Martin Mbonye Kayitale, An Hotterbeekx, Richard Idro, Issa Makumbi, Tom Lakwo, Bernard Opar, Joice Kaducu, Joseph Francis Wamala, Robert Colebunders

**Affiliations:** 1 Busitema University, Mbale, Uganda; 2 Infectious Disease Institute, Makerere University, Kampala, Uganda; 3 Global Health Institute, University of Antwerp, Antwerp, Belgium; 4 Department of Population Studies, School of Statistics, Makerere University, Kampala, Uganda; 5 Department of Paediatrics and Child Health, College of Health Sciences, Makerere University, Kampala, Uganda; 6 Ministry of Health, Kampala, Uganda; 7 World Health Organization, Juba, South Sudan

**Keywords:** Nodding syndrome, Epilepsy, Onchocerciasis, Prevalence, Incidence, Ivermectin, Larviciding, Uganda

## Abstract

**Background:**

Around 2007, a nodding syndrome (NS) epidemic appeared in onchocerciasis-endemic districts of northern Uganda, where ivermectin mass distribution had never been implemented. This study evaluated the effect of community-directed treatment with ivermectin (CDTI) and ground larviciding of rivers initiated after 2009 and 2012 respectively, on the epidemiology of NS and other forms of epilepsy (OFE) in some districts of northern Uganda.

**Methods:**

In 2012, a population-based community survey of NS/epilepsy was carried out by the Ugandan Ministry of Health in Kitgum and Pader districts. In August 2017, we conducted a new survey in selected villages of these districts and compared our findings with the 2012 data. In addition, two villages in Moyo district (where CDTI was ongoing since 1993) served as comparative onchocerciasis-endemic sites in which larviciding had never been implemented. The comparison between 2012 and 2017 prevalence and cumulative incidence were done using the Fisher’s and Pearson’s Chi-square tests at 95% level of significance.

**Results:**

A total of 2138 individuals in 390 households were interviewed. In the selected villages of Kitgum and Pader, there was no significant decrease in prevalence of NS and OFE between 2012 and 2017. However, the cumulative incidence of all forms of epilepsy decreased from 1165 to 130 per 100 000 persons per year (*P* = 0.002); that of NS decreased from 490 to 43 per 100 000 persons per year (*P* = 0.037); and for OFE from 675 to 87 per 100 000 persons per year (*P* = 0.024). The median age of affected persons (NS and OFE) shifted from 13.5 (IQR: 11.0–15.0) years in 2012 to 18.0 (IQR: 15.0–20.3) years in 2017; *P* <  0.001. The age-standardized prevalence of OFE in Moyo in 2017 was 4.6%, similar to 4.5% in Kitgum and Pader.

**Conclusions:**

Our findings support the growing evidence of a relationship between infection by *Onchocerca volvulus* and some types of childhood epilepsy, and suggest that a combination of bi-annual mass distribution of ivermectin and ground larviciding of rivers is an effective strategy to prevent NS and OFE in onchocerciasis-hyperendemic areas.

## Background

Nodding syndrome (NS) is a neurological disorder that manifests with a unique epilepsy type characterized by repeated head nodding, often in association with progressive neurocognitive impairment and physical decline [1–3]. It has been suggested that NS should be considered as one of the clinical presentations of onchocerciasis-associated epilepsy (OAE) [4, 5]. However, the pathophysiology of how *Onchocerca volvulus* may cause epilepsy remains unknown. Most cases of NS have been described in the onchocerciasis-endemic regions in northern Uganda [3, 6], western Uganda [7], South Sudan [8], and the Mahenge area in Tanzania [9]. More recent studies have reported nodding seizures among persons with epilepsy (PWE) in the onchocerciasis-endemic regions in Cameroon [10] and the Democratic Republic of Congo [11]. A high prevalence of epilepsy has also been reported in many onchocerciasis meso- and hyper-endemic regions [12–15] particularly where transmission is poorly controlled [16–19]. A study in an onchocerciasis-endemic region in the Mbam valley in Cameroon showed that the risk of developing epilepsy increased with increasing *O. volvulus* microfilarial density [20].

**Fig. 1:**
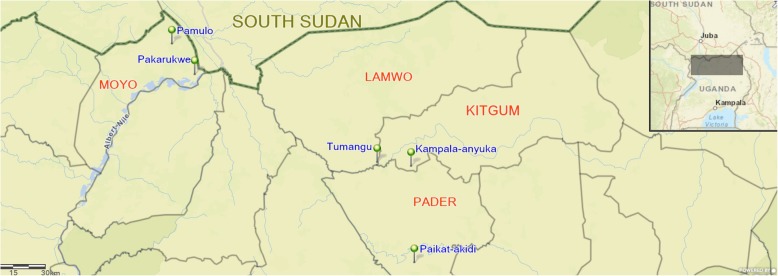
Map of northern Uganda showing the 2017 study sites

In Uganda, a NS epidemic occurred in the three onchocerciasis-endemic districts of Kitgum, Pader, and Lamwo in the northern part of the country [1] (Fig. 1).

These three districts were among the most affected by the Lord’s Resistance Army (LRA) civil war which took place between 1986 and 2006, and consequently hosted the highest number of Internally Displaced Persons (IDP) lodged in camps [21]. Several of these camps were located in onchocerciasis-endemic areas in close proximity to blackfly breeding sites. Due to the LRA insurgency, efforts to assess or control onchocerciasis in the area were compromised by insecurity (Table 1). By the year 2000, the mapping of onchocerciasis-affected areas had been completed in all three districts and consistent annual community-directed treatment with ivermectin (CDTI) started in 2009. Biannual CDTI was implemented in 2013. The average CDTI coverage in northern Uganda progressively improved from 33% in 2011 to 70% after 2015 [22]. In December 2012, the Government of Uganda launched vector control programs consisting of periodic ground larviciding of blackfly breeding areas in the three districts [22–24]. Aerial spraying was done as a one-off activity along the rivers Pager, Aswa and Agago which run through Kitgum, Pader and Lamwo districts resulting in a dramatic decrease of the blackfly population [24].

**Table 1:** A timeline of onchocerciasis control activities, carried out in Kitgum, Pader and Moyo districts

Year	Kitgum and Pader districts	Moyo district
1986–2006	Lord’s Resistance Army civil war interrupting onchocerciasis control efforts and programs	
1993	Partial mapping of onchocerciasis	Start of annual CDTI
1994–2008	Ivermectin only passively distributed	
2008	Onchocerciasis mapping completed	
2009	Start of consistent annual CDTI	
2012	Start of vector control (river larviciding + aerial spraying)	
2013	Start bi-annual CDTI	Start of bi-annual CDTI

*CDTI* Community-directed treatment with ivermectin

Since 2012, the number of new NS cases declined rapidly, with no new cases reported altogether since 2013 [25]. However, it is not known whether the incidence of other forms of epilepsy (OFE) also decreased in the same region.

Given that the number of new NS cases have been declining since 2012 [25], we hypothesized that following the implementation of onchocerciasis control measures in the districts of Kitgum and Pader, the incidence of OFE decreased alongside that of NS. In addition, we compared the 2017 prevalence of epilepsy in Kitgum and Pader with the prevalence of epilepsy in Moyo, a neighbouring onchocerciasis-endemic district where CDTI had been implemented since 1993 but without vector control activities.

## Methods

### Study sites

The study was carried out in three northern Uganda districts of Kitgum, Pader, and Moyo (Fig. 1). Kitgum stretches northwards from the northern border of Pader district to the southern border of South Sudan. Moyo is located in the North-Western part of Uganda, with river Nile forming its eastern border, and South Sudan on its northern border. The three districts have similar environmental characteristics, marked by bushy vegetation and an extensive network of water bodies. The population is settled in clusters of extended family homesteads with subsistence farming as their main activity.

### Study procedures

#### The 2012 survey in Kitgum, Pader and Lamwo districts

In July 2012, the Uganda Ministry of Health with local and international partners (World Health Organization [WHO], Centers for Disease Control and Prevention in the United States [CDC], African Field Epidemiology Network [AFENET], Makerere School of Public Health [MakSPH], etc.) carried out a house-to-house survey in all the villages of Kitgum, Pader and Lamwo districts, to establish the prevalence and epidemiological distribution of NS and epilepsy. These districts were purposively selected because they were the most affected by the NS epidemic, based on the national surveillance health reports. Village leaders carried out a census of all households in the village assisted by Village Health Teams (VHT). VHT are community volunteers who are identified by their community members and are given basic training by the Uganda Ministry of Health on major health programs so that they can mobilize and sensitize communities to actively participate in and utilize the available health services, including mass drug administration for neglected tropical diseases. Prior to the 2012 survey, these VHT were trained to identify NS and epilepsy cases using simplified community-based definitions [1]. The VHT in conjunction with the village leaders visited each household in their respective village and inquired from the household head or a responsible adult if there were any household residents suspected of having NS or OFE including the deceased cases. They used a simplified paper-based tool to collect this information (see Additional file 1). The community case definition of NS was: any person with observed or reported episodes of head nodding (“luc luc”, local word for NS), and that of epilepsy as any person with epileptic seizures (“olili” or “lili” or “cimu”, local word for OFE). Differentiation of NS and OFE was taught to the VHTs through dramatization of commonly observed symptoms like; muscle jerks and contractions, mouth-frothing, loss of sphincter control and loss of consciousness. If a person presented with nodding seizures but also muscle jerks and contractions, mouth-frothing, loss of sphincter control and loss of consciousness this person was considered to have both “luc luc” and “lili”.

As a follow-up of the 2012 survey described above, the CDC in conjunction with the Ugandan Ministry of Health (MOH) conducted a single-stage-cluster survey in March 2013. The aim of this survey was to systematically assess and validate the prevalence of NS cases in northern Uganda [26]. Thirty parishes were selected by single-stage cluster sampling with probability proportional to size; 20–30 children (aged 5–18 years) with reported head nodding were selected per parish using simple random sampling without replacement. VHT called selected persons and their caregivers to a central meeting point at a specified date and time. Trained and supervised local clinicians subjected the children to a standardized tool that applied the 2012 consensus case definition of NS [1, 26].

#### The 2017 survey in Kitgum, Pader, and Moyo

In August 2017, a house-to-house survey was carried out in selected villages of Kitgum, Pader and Moyo districts. Villages in Kitgum and Pader were randomly selected from the parishes with the highest prevalence of NS during the 2012 survey. The reason for selecting these high prevalence parishes was to allow us to demonstrate the effect of the onchocerciasis elimination measures with a relatively small population sample size. The parishes selected in Kitgum were: Lamit (Tumangu village) and Okidi (Kampala-Anyuka village). In Pader, Angole parish (Paikat-Akidi village) was selected. Given that the 2012 prevalence of NS in the parishes of Lamwo district was much lower (maximum of 2.9%) than most parishes in Kitgum and Pader, we did not include villages of Lamwo in the 2017 survey. In Moyo district, the villages of Pakarukwe and Pajakiri-North were randomly selected from among onchocerciasis-endemic parishes, as reported by the national health management information system (HMIS).

A two-stage epilepsy diagnosis method was used. Firstly, like in 2012, the trained VHT carried out a complete house-to-house screening of the entire village, during which demographic data was collected and suspected cases of NS and other forms of epilepsy identified. A household was defined as family members who ate from a common pot. The VHT asked the head of the household or any responsible adult present, the same two questions that were asked during the 2012 survey i.e. whether a family member was affected by “luc luc” (NS) or “lili” (OFE). Additionally, the VHT administered a locally adapted and pre-tested 5-question screening data collection tool for epilepsy [27]. The five questions were: 1) Loss of consciousness with either urine on self and/or drooling; 2) Absence(s) or loss of contact with the surrounding of sudden onset and of brief duration; 3) Jerking or uncontrolled abnormal movement (convulsion) of the limb(s) of sudden onset and lasting for a few minutes; 4) Sudden onset of brief, strange body sensations, hallucinations or illusions, be they visual, auditory or olfactory; 5) Previously told that he/she has epilepsy [27]. In Moyo, the NS question was not asked by the VHT because a local word for NS did not exist. However, the investigating clinicians questioned all suspected epilepsy cases about a history of nodding seizures; this was done in all study sites including Moyo.

Any household member who answered positively to at least one screening question was suspected to have epilepsy. During the second stage, persons suspected to have epilepsy underwent a comprehensive clinical and neurological assessment by physicians or trained clinical officers to confirm or reject the diagnosis of epilepsy and to specify the seizure type. All consenting persons with probable NS or OFE were finger-pricked to test for the presence of *O. volvulus*-specific Ov16 IgG4 antibodies using rapid diagnostic tests (RDT) (Standard diagnostics Inc., Gyeonggi-do, Republic of Korea).

### Definitions

A case of **epilepsy** was a person who had experienced at least two unprovoked seizures with a minimal time difference of 24 h between the two events [28].

A case of **NS** was defined as a person with head nodding seizures (repetitive involuntary drops of the head towards the chest on two or more occasions) as confirmed by a trained clinician, that occurs in a previously healthy child [1].

A case of **OFE** was a case of epilepsy, without a history of nodding seizures.

### Data management

All data collection tools used during the 2012 and 2017 survey are available as Additional file 1. During the 2017 survey, VHT collected household survey data on paper which was then electronically captured using the software Epi-Info™ (CDC, Atlanta, USA) and then transferred to Microsoft Excel 2016 (Microsoft, Seattle, USA) for further cleaning. Physicians collected data on persons suspected to have epilepsy on electronic tablets via questionnaires developed on the Open Data Kit platform (https://opendatakit.org/). The collected data was further processed in Microsoft Excel 2016 and exported to Stata version 13 (StataCorp LLC, College Station,Texas, USA) for statistical analysis.

### Statistical analysis

#### 2012 prevalence of NS and OFE in Kitgum and Pader

We calculated the 2012 epilepsy prevalence using the population estimates of the selected villages during that period [29, 30]. Age-specific prevalence was obtained using the age structure obtained for each village during the 2017 survey.

#### 2017 Prevalence of NS and OFE in Kitgum, Pader, and Moyo

For the 2017 survey, crude estimates for NS and OFE prevalence were calculated by dividing the number of confirmed cases by the total population of the households visited during the survey. For better comparison, differences in the age distribution of the study populations were overcome by applying direct standardization on the crude age-specific prevalence rates. We used the population age structure (age groups: 0–9, 10–19, 20–29, 30–34, 35–39, 40–44, ≥ 40 years) reported by Uganda Housing and Population Census as a reference population [31]. To calculate the age-standardized prevalence of NS and OFE per village, we used the 2017 Uganda national bureau of statistics (UBOS) projected mid-year population estimates for each study village [31].

We calculated the sensitivity and specificity of a positive answer to the questions “luc luc” and “lili” for a confirmed diagnosis of respectively NS and OFE. Given that no confirmation of “luc luc” and “lili” diagnosis was done in 2012, and as the same VHT were involved in 2012 and the 2017 surveys, the sensitivity/specificity of the “luc luc” and “lili” diagnoses obtained during the 2017 validation process were applied to correct the 2012 findings. The comparison between 2012 and 2017 prevalence was done using Fisher’s and Pearson’s Chi-square tests at 95% level of significance.

#### Cumulative incidence of NS and OFE

The cumulative incidence (or incidence proportion) of NS/OFE in Kitgum and Pader was estimated using data from the 2 yr preceding the 2012 and 2017 surveys. For the period before biannual CDTI and river larviciding, the total number of NS with onset between 2011 and 2012 as revealed by the 2012 survey was halved to obtain the average number of incident cases per year. This was then divided by the total population of the study sites as of 2012. Similarly in 2017, we summed the number of cases with onset between 2016 and 2017, divided by two to obtain the average number of incident cases per year, and used the 2017 survey population as the denominator. We assumed a stable village population for the 2 yr used to calculate incidence. The cumulative incidence was expressed per 100 000 persons per year. Given that cumulative incidence measures proportions, comparisons were done using the Chi-squared test.

## Results

### The 2012 household survey in Kitgum and Pader district

**Fig. 2:**
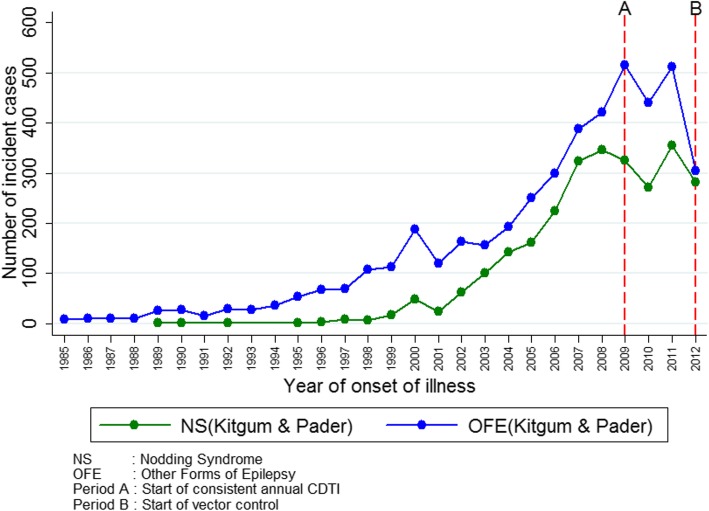
Number of new NS and OFE cases per year according to the 2012 community census in Kitgum and Pader districts

The number of new OFE cases every year had been relatively stable until the late 1990’s when there was a sharp rise that peaked in 2007, followed by a decline in new cases after 2008 (Fig. 2). The earliest NS cases according to the 2012 data appeared in 1989; after 1998, the number of new NS cases began to rise in a similar time pattern as OFE cases.

### The 2017 household survey in Kitgum, Pader, and Moyo

In the three districts of Kitgum, Pader, and Moyo, a total of 390 households were visited from which 2138 persons were screened for epilepsy (Table 2).

**Table 2:** Household and individual characteristics of the 2017 epilepsy survey (Kitgum, Pader and Moyo districts)

	Kitgum	Pader	Kitgum & Pader	Moyo
Household characteristics				
Number of households: *n* (%)	142 (36.4)	55 (14.1)	197 (50.5)	193 (49.5)
Median household size: *n* (IQR)	6 (4–8)	6 (4–8)	6 (4–8)	5 (3–7)
Agriculture as main activity: *n* (%)	134 (94.4)	54 (98.2)	188 (98.9)	184 (95.3)
Family history of death from epilepsy: *n* (%)	15 (10.6)	2 (3.36)	17 (8.6)	7 (3.6)
Age (years) at death of PWE^a^: median (IQR)	16 (13–19)	33 (22–44)	16 (13–19)	17 (14–28)
Study population				
Number of participants: *n* (%)	861 (40.2)	316 (14.8)	1177 (55.1)	961 (45.0)
Age: median (IQR)	16 (8–29)	17 (7–26)	16 (7–28)	16 (8–30)
Male gender: *n* (%)	419 (50.5)	163 (53.4)	582 (51.3)	442 (48.2)
Ivermectin use in 2017: *n* (%)	716 (83.5)	249 (79.6)	965 (82.5)	779 (81.2)
Age distribution (years): *n* (%)				
0–9	258 (29.8)	98 (31.0)	356 (30.3)	279 (29.0)
10–19	260 (30.2)	89 (28.2)	349 (29.7)	268 (27.9)
20–29	131 (15.2)	61 (19.3)	192 (16.3)	154 (16.0)
30–39	77 (8.9)	20 (6.3)	97 (8.2)	93 (9.7)
≥ 40	135 (15.7)	48 (15.2)	183 (15.6)	167 (17.9)
Confirmed persons with epilepsy				
Number of PWE^a^:	83	29	112	46
Male gender: *n* (%)	49 (59.0)	17 (58.6)	66 (58.9)	21 (45.7)
Female gender: *n* (%)	34 (41.0)	12 (41.4)	46 (41.1)	25 (54.3)
Age in years: median (IQR)	19 (16–22)	16 (14–18)	18 (16–20)	23 (15–36)
Age at seizure onset (years): median (IQR)^b^	9 (7–12)	8 (7–11)	9 (7–12)	10 (2–15)
Year of onset of seizures: median (IQR)^b^	2007 (2005–2010)	2010 (2009–2012)	2008 (2006–2011)	2009 (2000–2012)
Positive Ov16 test: *n* (%)^c^	13 (27.7)	8 (50.0)	21 (33.3)	16 (36.4)

*IQR* Interquartile range

^*a*^*PWE* Persons with epilepsy, including those with nodding seizures

^b^27 missing

^c^Only a limited number of persons were tested (test not available or participant declined to be tested)

More than 95% of the households in every selected village participated. A total of 1598/2138 (74.7%) participants were aged below 30 years; the median age was 16 years (Interquartile Range [IQR]: 14–49). Death of a family member with epilepsy was reported by 21 (5.6%) households (24 deaths in 21 households). Overall, 1746 (81.7%) participants reported having taken ivermectin during the last mass distribution (April 2017). Of the household members screened, 163 were suspected to have epilepsy and the diagnosis was confirmed in 158/163 (96.9%) of them (Table 2). Of the five individuals not confirmed to have epilepsy, four were diagnosed as febrile seizures and one as mentally disabled.

The median age of PWE was 19 years (IQR: 16–23), and the median age at onset of seizures was 9 years (IQR: 6–14). The most frequent seizure type, other than NS, was generalized tonic-clonic seizures. Among the PWE who had consented to Ov16 testing, the percentage of Ov16 seropositivity was 27.7% in Kitgum, 50.0% in Pader, and 36.4% in Moyo. Nine (32.1%) of the 28 persons with NS were Ov16 seropositive compared to 28/79 (35.4%) persons with OFE.

Based on 1147/1177 (97.5%) household members and 81/112 (72.3%) confirmed cases of NS/epilepsy in Kitgum and Pader who had complete information, we assessed the performance of the “luc luc” and “lili” questions for the diagnosis of confirmed NS/epilepsy as follows: sensitivity is 98.8% (80/81) and specificity is 97.0% (1034/1066). (The detailed characteristics of each screening question in the discrimination between NS and OFE are summarized in Table 3.

**Table 3:** Performance of the screening questions “luc luc” and “lili” in diagnosing NS and OFE during the 2017 survey

	Yes to “luc luc”^a^ or “Lili”^b^	Yes^c^ to “luc luc” only, or to both “luc Luc” and “Lili”	Yes to “lili” only
Confirmed nodding syndrome: *n* (%)	38 (46.9)	33 (63.5)	5 (17.2)
Other forms of epilepsy: *n* (%)	42 (51.9)	18 (34.6)	24 (83.8)
Non-epileptic^d^: *n* (%)	1 (1.2)	1 (1.9)	0
Total	81	52	29

^a^Local term for nodding syndrome

^b^Local term for other forms of epilepsy

^c^Includes a history of nodding syndrome, with or without other forms of seizures (NS and NS+)

^d^Suspected case, but not confirmed as epilepsy

*NS* Nodding Syndrome only, *NS* plus Nodding Syndrome and other seizures, *OFE* Other Forms of Epilepsy

### Prevalence of NS and OFE in 2017 in Kitgum, Pader, and Moyo

The highest crude prevalence of NS was observed in Pader (5.1% in Paikati-Akidi village). In Kitgum district, the crude prevalence of NS was 4.6 and 4.4% in Tumangu and Kampala-Anyuka villages respectively. On the other hand, OFE were more prevalent in the Kitgum district (7.8% in Tumangu and 4.2% in Kampala-Anyuka) compared to Pader (4.1% in Paikati-Akidi village). The 10–19 years age group was most affected by NS, while OFE were more frequent among the 20–29 years old. The prevalence of epilepsy in Moyo was 4.8%, and no nodding seizures were reported. The difference in the age-specific prevalence of OFE between Moyo and other study sites was most significant in the 10–19 years age group (*P* = 0.005) (Table 4).

**Table 4:** The crude and age-specific prevalence of nodding syndrome and other forms of epilepsy in the 2017 survey, in Kitgum, Pader and Moyo districts

	Kitgum *n* = 861	Pader *n* = 316	Kitgum & Pader *n* = 1177	Moyo *n* = 961	*P*-value
NS and NS plus cases only					
Crude prevalence of NS: %	4.3	5.1	4.5	0	NA
95% confidence interval of prevalence	3.1–5.9	3.0–8.3	3.4–5.9	0	
Age-standardized prevalence rate of NS: %	3.7	4.4	3.8	0	NA
Age-specific prevalence of NS: *n* (%)					
0–9 years	1 (0.4)	1 (1.0)	2 (0.6)	0	NA
10–19 years	27 (10.4)	14 (15.7)	41 (11.8)	0	NA
20–29 years	9 (6.9)	1 (1.6)	10 (5.2)	0	NA
30–39 years	0	0	0	0	NA
≥ 40 years	0	0	0	0	NA
OFE only					
Crude prevalence of OFE: %	5.3	4.1	5.0	4.8	0.831
95% confidence interval of prevalence	4.0–7.1	2.3–7.1	3.9–6.5	3.6–6.4	
Age-standardized prevalence of OFE: %	5.1	3.7	4.5	4.6	0.98
Age-specific prevalence of OFE: *n* (%)					
0–9 years	2 (0.8)	2 (2.0)	4 (1.1)	9 (3.2)	0.089^a^
10–19 years	15 (5.8)	8 (9.0)	23 (6.6)	5 (1.9)	***0.005***^***b***^
20–29 years	25 (19.1)	2 (3.4)	27 (14.1)	17 (11.1)	0.412^b^
30–39 years	1 (1.3)	1 (5.3)	2 (2.1)	4 (4.3)	0.442^a^
≥ 40 years	3 (2.2)	0	3 (1.6)	11 (6.6)	0.026^a^
All epilepsy cases (NS + OFE)					
Crude epilepsy prevalence: %	9.6	9.2	9.5	4.8	< 0.001
95% confidence interval of prevalence	7.8–11.9	6.3–13.1	7.9–11.4	3.6–6.4	
Age-standardized prevalence rate of epilepsy: %	8.8	7.9	8.3	4.6	< 0.001

*IQR* Interquartile range, *NS* Nodding Syndrome only, *NS plus* Nodding Syndrome and other seizures, *OFE* Other Forms of Epilepsy, *NA* Not applicable, *CI* Confidential interval

^a^Fisher’s exact chi-square test

^b^Pearson chi-square test

**Fig. 3:**
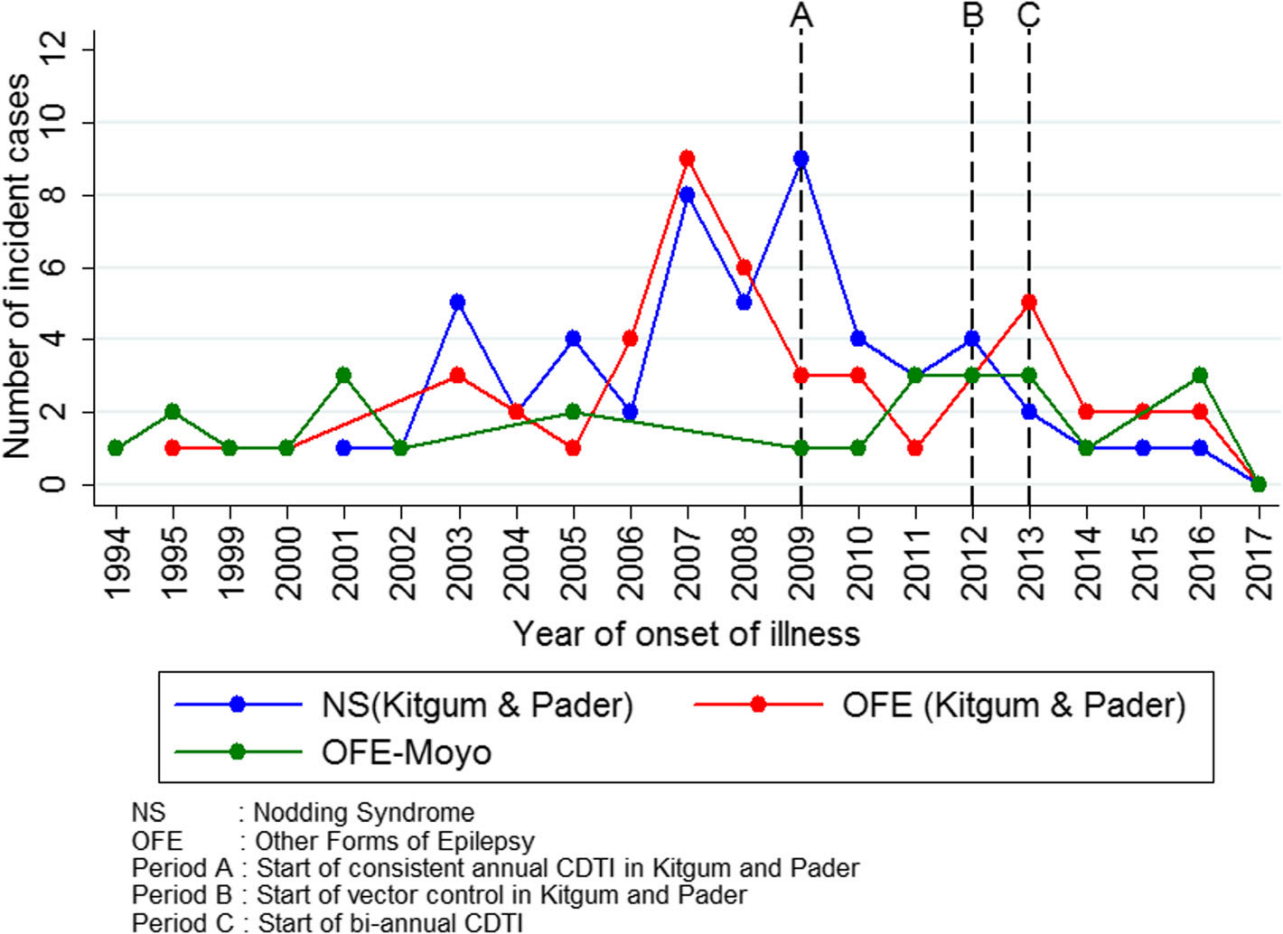
Number of new NS and OFE cases per year of onset in Kitgum, Pader and Moyo districts according to the 2017 survey

Using data collected during the 2017 survey, we observed that before 2007 there had been a gradual increase in new cases per year for both NS and OFE in Kitgum and Pader (Fig. 3). There was a peak in 2007 for OFE and in both 2007 and 2009 for NS. After 2013, there was a steady decline in the number of new cases of NS and OFE; eventually, no new case of NS was recorded for the year 2017.

### Comparison of prevalence and incidence of NS and OAE in selected villages in Kitgum and Pader districts between 2012 and 2017

Over a five-year period (2012–2017), the prevalence of NS and OFE did not change significantly in Kitgum and Pader. However, the cumulative incidence of all forms of epilepsy decreased from 1165 to 130 per 100 000 persons per year (*P* = 0.002); that of NS decreased from 490 to 43 per 100 000 persons per year (*P* = 0.037); and for OFE from 675 to 87 per 100 000 persons per year (*P* = 0.024) (Table 5). The number of new NS cases per year dropped from four in 2012, to zero in 2017 (Fig. 3).

**Table 5:** Comparison of crude prevalence and incidence of nodding syndrome and other forms of epilepsy in Kitgum and Pader districts during the 2012 and 2017

Selected villages	All forms of epilepsy			Nodding syndrome			Other forms of epilepsy		
	2012	2017	*P*-value	2012	2017	*P*-value	2012	2017	*P*-value
Prevalence in Kitgum district									
Kampala-anyuka: *n* (%)	54 (11.5)	47 (8.5)	0.110	22 (4.6)	24 (4.4)	0.798	32 (6.9)	23 (4.2)	0.061
Tumangu: *n* (%)	24 (10.1)	35 (12.3)	0.392	10 (4.2)	13 (4.6)	0.811	14 (5.8)	22 (7.8)	0.381
Prevalence in Pader district									
Paikati-akidi: *n* (%)	32 (11.9)	30 (9.5)	0.347	13 (4.8)	16 (5.1)	0.905	19 (7.0)	14 (4.4)	0.166
Kitgum and Pader districts combined									
Prevalence: *n* (%)	110 (11.2)	112 (9.7)	0.251	45 (4.6)	53 (4.6)	1.00	65 (6.6)	59 (5.1)	0.134
95% *CI* of prevalence	10.6–14.8	8.1–11.6		3.4–6.1	3.5–6.0		5.2–8.4	3.9–6.6	
Cumulative incidence^a^	1165	130	0.002	490	43	0.037	675	87	0.024

2012 population distribution of: Kampala-anyuka: *n* = 469, Tumangu: *n* = 241, Paikati-akidi: *n* = 269

2017 population distribution of: Kampala-anyuka: *n* = 552, Tumangu: *n* = 284, Paikati-akidi: *n* = 317

All comparisons done using Pearson chi-square test

*CI* Confidential interval

^a^Cumulative incidence per 100 000 persons per year

### Comparison of ages of persons with NS and OFE in Kitgum and Pader districts between 2012 and 2017

**Fig. 4:**
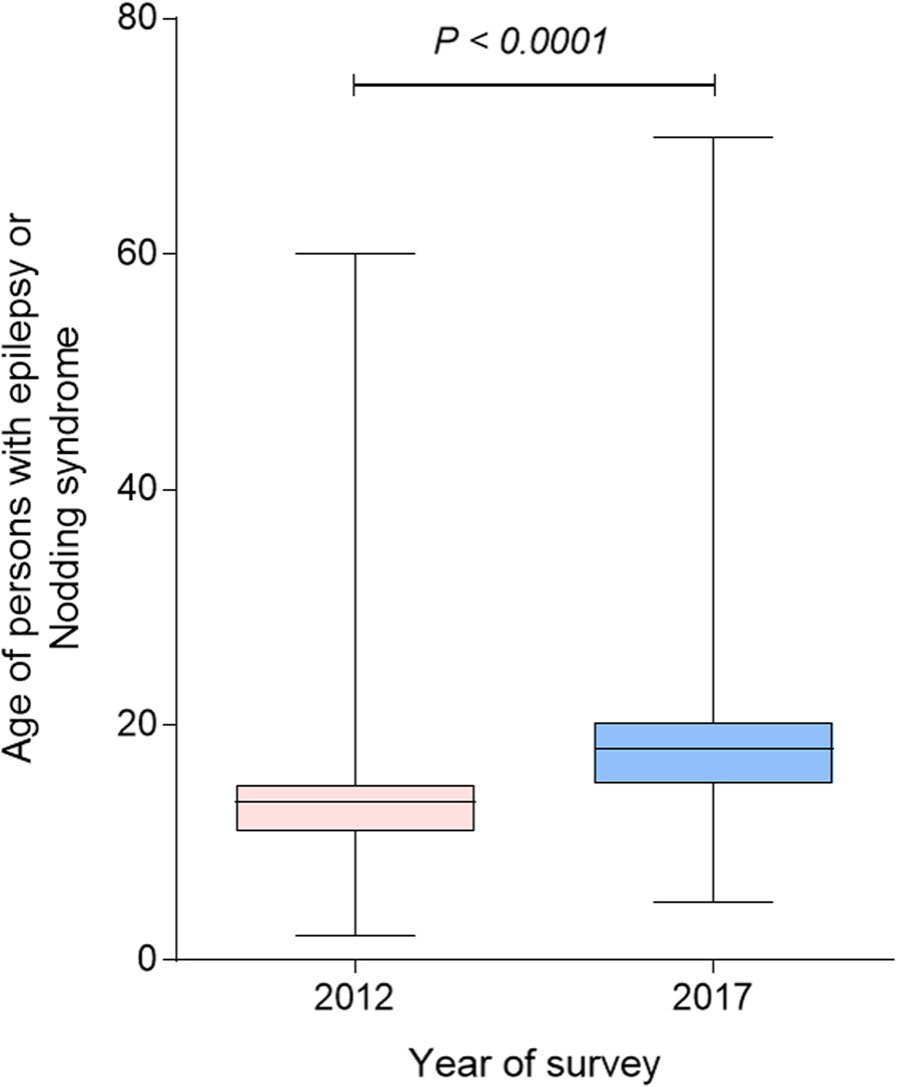
Age of persons with NS and OFE identified during the 2012 and 2017 surveys in Kitgum and Pader

Compared to the 2012 data, there was an age shift of PWE to older age groups in 2017. The median age of all persons with epilepsy shifted from 13.5 years (IQR: 11.0–15.0) in 2012 to 18.0 years (IQR: 15.0–20.3) in 2017; *P* < 0.001 (Fig. 4).

## Discussion

The 2012 epilepsy survey shows that in the absence of onchocerciasis elimination measures, a simultaneous sharp increase in NS and OFE cases could occur as was observed in Kitgum and Pader (Fig. 2). We discussed the potential reasons for this increase in a previous publication [32]. Most likely, they are multifactorial and include the close proximity of IDP camps to the blackfly breeding sites, coupled with no access to ivermectin. Other factors that may have played a role could be the poor nutritional status of the children during the war, making them susceptible to more severe *O. volvulus* infection as well as other health conditions. Another event that occurred prior to the NS epidemic was the theft of about 300 000 cows from the Acholi people in northern Uganda [33]. This is relevant for onchocerciasis because these *Onchocerca ochengi*-infected cows may have played a protective role against human *O. volvulus* infection in these villages in the past [34].

Our 2017 epilepsy survey shows the effects of optimal onchocerciasis elimination interventions (aerial spraying of breeding sites, ground larviciding of rivers, and bi-annual ivermectin distribution) on the incidence and prevalence of both NS and OFE. In 2017, the prevalence of epilepsy in Kitgum and Pader remained high. This was expected because 5 yr of optimal onchocerciasis control may not be enough to alter the prevalence of OAE, especially if PWE is treated with anti-epileptic drugs such that they survive and therefore do not exit the patient pool for several years.

The high prevalence of epilepsy in Moyo is not surprising because the villages included in this study were also located in an onchocerciasis-endemic region. Despite long-standing annual, then later bi-annual distribution of ivermectin in Moyo, the number of new epilepsy cases remained relatively stable every year. The average CDTI coverage in Moyo in the past decade has been greater than 85% [24], and the 2017 Ov16 seroprevalence among PWE in Moyo was 36.4%. Whether ongoing onchocerciasis transmission could be the cause of the persistently high epilepsy incidence observed in this district needs to be investigated. The medical history of most PWE excluded a number of common causes of epilepsy such as perinatal anoxia, brain trauma, cerebral malaria, and meningitis. It is possible however that neurocysticercosis was responsible for part of this high epilepsy prevalence.

In contrast to villages Moyo district, there was a steady decline of new epilepsy cases in Kitgum and Pader following the introduction of annual CDTI in 2009 and a further decrease after river larviciding was initiated in 2012. This suggests that in areas of high onchocerciasis endemicity, the combination of bi-annual ivermectin distribution and ground larviciding of rivers may offer better and faster protection of the population against OAE because it results in a rapid decrease of the microfilarial load in the population. Contrary to previous reports from the Ugandan Ministry of Health [25], we encountered 3 NS cases who reported an onset of the seizures after 2013. Potentially, a recall bias concerning the exact year of onset of NS could explain these late-onset cases.

The age shift observed in persons with NS and OFE to older age groups is most likely explained by a decreased incidence of OAE in the 5–18 years old age group and an increased survival because of increased access to anti-epileptic treatment and better nutrition. Similar epidemiological trends of NS and OFE suggests that both conditions may be sharing the same risk factors and etiological agent. The Ov16 seroprevalence obtained using the rapid test was similar among persons with NS and OFE, and much lower than the 66.7% Ov16 ELISA prevalence reported among NS cases in the same area in 2009 [6]. The low Ov16 prevalence in 2017 corroborates with the findings of Weil et al. which showed a reduced sensitivity of the Ov16 rapid tests especially after treatment with ivermectin [35]. The low level of Ov16 prevalence among OFE cases is also explained by the fact that in 2017 less PWE presented with OAE.

Our study illustrates the importance of strengthening onchocerciasis elimination programs, particularly in areas with high epilepsy prevalence. Recent epilepsy surveys in Cameroon and Tanzania have shown that annual CDTI has only a limited effect on the incidence of OAE [19, 36]. A recent prospective study in Cameroon also showed that the risk to develop epilepsy depends on the microfilarial load during childhood [20]. While it is known that the microfilarial load in an infected individual tends to increase with age, it has also been reported that children develop NS at an earlier age than OFE, while onchocercal blindness is observed in general only after the age of 20 years. This suggests that the microfilarial threshold to develop epilepsy is lower than for blindness; it also explains why annual CDTI may have decreased onchocercal blindness dramatically but does not seem to decrease the community microfilarial load enough to substantially impact the incidence of epilepsy.

While our study supports the growing epidemiological evidence that infection with *O. volvulus* may be directly or indirectly associated with epilepsy, the pathophysiological mechanism of how this may happen still needs to be elucidated. A recently published post-mortem study of five persons from northern Uganda who died of NS suggested that NS is a tauopathy and a neurodegenerative disease [37]. However, these histopathological findings are most likely the consequence of NS rather than its cause. Indeed, we carried out another post-mortem study in the same area, among five persons with NS and four persons with another type of OAE. This study revealed similar neuro-inflammatory histopathological changes with mild to sparse deposits of tau-immunoreactive neurofibrillary tangles in 4/5 persons with NS and 2/4 persons with another form of OAE, suggesting that NS and other forms of OAE are caused by a similar phenomenon [38]. The tau deposits most likely are induced by repetitive seizures [39] with seizure-associated hypoxia [40] and possibly repeated head injuries [41]. The decreasing incidence of both NS and OFE after strengthening onchocerciasis elimination measures suggests that both conditions may be *O. volvulus*-related.

The strength of our study is that it is a population-based study comparing the same villages over time and comparing two different onchocerciasis foci exposed to different onchocerciasis control measures. However, our study also has several limitations. The 2012 and 2017 survey used slightly different methodologies, complicating the comparison of the prevalence and incidence data. Moreover, the diagnosis of epilepsy was mainly made by clinical officers or medical doctors but was not confirmed by a neurologist. In addition, no laboratory investigations were performed besides the Ov16 testing in some participants. Other causes of epilepsy were only excluded by medical history. The incidence of epilepsy in our study was based on interviewing PWE and not prospectively by an epilepsy surveillance system. A setback of this approach is the introduction of recall bias, especially regarding the year of epilepsy onset for PWE who had died before the surveys. Therefore our incidence data becomes less reliable as the year of onset is further in the past. Another limitation is the fact that a different team of clinical officers participated in the survey in Moyo; however, they had received the same training as those who investigated Kitgum and Pader districts and were supervised by the same medical doctor (NG).

Because there was no local word for NS in Moyo, it is possible that some NS cases could have gone unrecognized. In 2012, in the high epilepsy prevalence villages in Kitgum and Pader, the prevalence of NS might have been overestimated. Indeed, because of national and international attention about NS, children with OFE could have been reported by family members to have NS in order to attract more support and interest of funders (Wamala J, personal communication). Our 2017 survey results showed that 34.6% of the “luc luc” screened by the VHT were not confirmed to be NS cases but rather as OFE. This figure is only slightly lower than the 45% reported in the CDC/MOH validation study performed in 2013 [26]. The high specificity and sensitivity of the questions “luc luc” or “lili” for screening PWE, and the fact that these questions were asked during both surveys, increases the reliability of our findings when considering the overall prevalence and incidence of epilepsy (including NS) between 2012 and 2017.

## Conclusions

Our study illustrates the importance of the public health problem, not only caused by NS but also by OFE in places where onchocerciasis elimination measures are not implemented or are sub-optimal. The lesson learned from the epilepsy epidemic in northern Uganda is that onchocerciasis elimination strategies need to be revised and retargeted, taking into account OAE.

## Supplementary information

**Additional file 1.** 2012 Village Health Team (VHT) nodding syndrome case report form.

## Data Availability

All collected data is confidentially kept at both the Global Health Institute, University of Antwerp (Belgium) and the Infectious Disease Institute in Kampala (Uganda). The datasets are available from the corresponding author on a reasonable request.

## Abbreviations

AFENET: African Field Epidemiology Network
CDC: Centers for Disease Control and Prevention
CDTI: Community directed treatment with ivermectin
IDP: Internally displaced persons
IQR: Interquartile pange
LRA: Lord’s Resistance Army
MakSPH: Makerere School of Public Health
MOH: Ministry of Health
NS: Nodding syndrome
NS +: Nodding syndrome plus.
OFE: Other forms of epilepsy
RDT: Rapid diagnostic test
VHT: Village health teams
WHO: World Health Organization
