# Supplementary material for: Prevalence and incidence of nodding syndrome and other forms of epilepsy in onchocerciasis-endemic areas in northern Uganda after the implementation of onchocerciasis control measures
Source: Infect Dis Poverty. 2020 Mar 2;9:12. doi: 10.1186/s40249-020-0628-3 (PMC7050130; doi:10.1186/s40249-020-0628-3)
Supplement: Supplementary file 1 — Additional file 1. 2012 Village Health Team (VHT) nodding syndrome case report form. [file 40249_2020_628_MOESM1_ESM.pdf]

ADDITIONAL FILE 1

2012 Village Health Team (VHT) nodding syndrome case report form

Date: ……………………..District: …………………………… Sub-county:……………….
Parish: ………………………. Village: ………………………………..
Name of LCI Chair: ……………………………………… Phone: …………………………

Name of Household head: ……………………………………………
Phone number of Household head: …………………………

Serial No.	Name of patient	Age [Yrs]	Sex [M/ F]	Type of Disease (Yes or No)		Date of onset (month/ yr)	Case Status Alive [A]; or Dead [D]
				Nodding Syndrome *("Luc luc")*	Epilepsy *("Lili")*		

Village: ………………

Name of VHT ………………………. Phone: ……………………

Signature: …………………………..

Use one form per household. Start by listing the nodding syndrome and/ or epilepsy cases that are alive. Then inquire and list all household members who had either nodding syndrome and/ or epilepsy but are dead.

2017 survey: Household level questionnaire

NOTE: This questionnaire will be administered in a digitalized form on a tablet computer that will be used as a data collection tool during this survey. Within each household, all participants will be asked all the questions in sections 1.2 and 1.3 during one single interview sequence.

DATE: _____ / _____ / ________

FULL NAME OF INTERVIEWER: ____

DISTRICT: ____

PARISH: ____

VILLAGE: ____

1.1. HOUSEHOLD CHARACTERISTICS

UNIQUE HOUSEHOLD CODE:

XX /XX /XX/XXXX (First 2 letters of: District/Parish/Village, followed by number (e.g.0001))

GPS coordinates of household:

LATITUDE __ __. __ __ __ __ __LONGITUDE __ __. __ __ __ __ __ ALTITUDE _ __ __ __

Full name of household head: ____

Mobile phone number of household head: ____

Ethnic group of household head: ____

Total number of people in the household: ____

Livelihood generating activities of the family: ☐ Farmer ☐ Livestock breeder ☐ Fisherman ☐ Civil service ☐ Business / trader ☐ Craftsman ☐ other, specify:_____________________

Has there been a family member that died from any form of epilepsy or nodding syndrome, lili or luc luc?

☐YES ☐NO ☐DON'T KNOW

IF YES: When (year) ________ At what age: ________
years
IF MORE THAN ONE: When (year) ________ At what age: _____ years

When (year) ________ At what age: _____ years

Did the family or any family member(s) participate in the 2012 and / or 2013 survey on NS?
☐YES ☐NO ☐DON'T KNOW

1.2. INDIVUDUAL INTERVIEW WITH EACH HOUSEHOLD MEMBER

Person ID: ____

Full Name: ____

Age: _years
Birth date: ___/___/____
Sex: ☐Male ☐Female

Is the person present during the interview visit? ☐YES ☐NO
Who is answering to the questions? ☐self ☐mother ☐household head
☐other, specify
____

1.3. SCREENING FOR EPILEPSY AND NODDING SYNDROME CASES

If at least one of the 8 questions is answered with YES, the electronic questionnaire will automatically report the person for invitation to participate in the neurological survey for case verification.

QUESTION A.
Is the participant / are you suffering from luc luc (nodding syndrome)?

☐YES ☐NO ☐DON'T KNOW

QUESTION B.
Is the participant / are you suffering from Lili (epilepsy)?

☐YES ☐NO ☐DON'T KNOW

QUESTION C.
Is the person known to suffer from both, luc luc (nodding syndrome) and Lili (epilepsy)?

☐YES ☐NO ☐DON'T KNOW

QUESTION 1.
Have you ever suddenly fallen to the ground and experienced:

a) Loss of consciousness ☐YES ☐NO ☐DON'T KNOW

b) Loss of bladder control? ☐YES ☐NO ☐DON'T KNOW

c) Foam at the mouth? ☐YES ☐NO ☐DON'T KNOW

QUESTION 2.
Have you ever experienced absence(s) or sudden loss(es) of contact with the surroundings, for a short duration of time?
☐YES ☐NO ☐DON'T KNOW

QUESTION 3.
Have you ever experienced sudden, uncontrollable twitching or shaking of your arms, legs or head, for a period of a few minutes?

☐YES ☐NO ☐DON'T KNOW

QUESTION 4.
Do you sometimes experience sudden and brief bodily sensations, see or hear things that are not there, or smell strange odours?

☐YES ☐NO ☐DON'T KNOW

QUESTION 5.
Have you ever been told that you are suffering from epilepsy or have you already experienced **at least one episode of seizures**?

☐YES ☐NO ☐DON'T KNOW

1.3. IVERMECTIN USE

Did you take ivermectin during the last distribution in 2016? ☐YES ☐NO ☐DON'T KNOW

Signature of Interviewer____

2017 survey: Individual Neurology questionnaire

DATE: _____ / _____ / ________

FULL NAME OF THE INTERVIEWER: ____

PARTICIPANT IDENTIFICATION

Participant ID ____
Last name *(in capital letters)*: ____
First name: ____

The medical doctor/neurologist will first assess whether the person referred to him has epilepsy (Nodding syndrome or another form of epilepsy)

Diagnosis of Nodding syndrome or another form of epilepsy is confirmed

☐YES ☐NO

If NO: other diagnosis?

☐ Recurrent febrile convulsions
☐ Dizziness / syncope
☐ Paroxysmal vertigo
☐ Severe anaemia
☐ Mental retardation without epilepsy
☐ Psychiatric illness without epilepsy
☐ Classic migraine
☐ Other, specify______________________

If YES the following neurology questionnaire will be administered in a digitalized from using tablet computers. By indicating the individual Participant ID on paper forms, the neurologist can provide additional important notes of the anamnesis on paper.

PARTICIPANT IDENTIFICATION

Participant ID :___________________________________

Last name *(in capital letters)*: ___________________

First name:____

Town / Village:____

District:____

Phone number:____

Sex: ☐ Male ☐ Female
Age: ________________

Date of birth: ___/___/________

Place of birth: ____

Country of birth:____

Ethnic group: ____

Marital status: ☐ Married ☐ Living with parents ☐ In partnership ☐ Living alone ☐ Other, specify ______________

Is the participant answering himself / herself? ☐ YES ☐ NO
If NO, who is answering and what is the relationship between the participant and respondent?

Full name:____

Relation to participant: ☐ Mother ☐ Father ☐ Sibling ☐ Other, specify ___________

DEMOGRAPHIC DATA

Since how long is the participant lives in the village? __________YEARS
IF less than 1 year: how many MONTHS? __ __

Was the interviewed participant living in the village in 2011-12?
☐ Yes ☐ No ☐ DON'T KNOW

IF NO, in which village has the participant lived before?
Village: _________________________________
Area: _________________________________
Health zone: _________________________________

IF in a foreign country: in which country has the participant lived before?____

Are you currently attending school? ☐ YES ☐NO

School grade level completed: ☐None
☐Primary ☐ P1 ☐P2 ☐P3 ☐P4 ☐P5 ☐P6 ☐P7
☐Secondary ☐ S1 ☐S2 ☐W3 ☐S4 ☐S5 ☐S6
☐Tertiary ☐ University ☐ Vocational/ college / Institute

IF NO: At which level did you stop school attendance: __________________________

Reason:
☐ Due to epileptic seizures while in school
☐ Fear to leave the epileptic child unattended
☐ Due to epilepsy related stigma
☐ Due to epilepsy related learning difficulties
☐ Other reasons (financial, other illness than epilepsy, accessibility,)
☐ DON'T KNOW

ADULT participant:
Occupation / livelihood activity: ☐ Domestic worker ☐ Farmer ☐ Livestock breeder ☐ Fisherman ☐ Civil servant ☐ Craftsman

☐ Student ☐ Business / trader ☐ none ☐ other, specify: _______________________

CHILD Participants:
Livelihood activity of family: ☐ Domestic worker ☐ Farmer ☐ Livestock breeder

☐ Fisherman ☐ Civil servant ☐ Craftsman ☐ Student ☐ Business / trader ☐ None
☐ other, specify:_________________________

HISTORY OF EPILEPSY AND HEAD NODDING

Nodding History
Does the participant have a history of head nodding (repetitive involuntary drops of the head towards the chest on 2 or more occasions): ☐YES ☐NO
IF YES: Year of onset ____________
IF YES:
Was the diagnosis of nodding syndrome made by a doctor? ☐YES ☐NO ☐DON'T KNOW
What is the mental status during the head nodding?
☐ Awake and still able to respond
☐ Awake but not responding
☐ Decreased consciousness, not responding
☐ Unconscious

How old (years) was the child when the head nodding started? ___________years

What triggers the head nodding? (Tick which ever applies)
☐ Spontaneous (no obvious trigger)
☐ Food ☐ Cold weather
☐ Nothing ☐ DON'T KNOW
☐ Other, specify _______________________________

Has head nodding continued until today? ☐ Yes ☐ No
IF YES:
What was the frequency of head nodding attacks in the last three months: (Tick highest appropriate frequency):
☐ less than1 episode per week
☐ 1 or more episodes per week
☐ At least 1 episode per day
(Approximate number: _______/day)

Is the participant currently on treatment for nodding syndrome? ☐ Yes ☐ No
IF YES: specify treatment: ____
If yes, where does the participant go for treatment?
☐ Designated treatment center (Name____________)
☐ NS outreach post
☐ Other, specify ______________________
IF the nodding stopped:
At what age the nodding stopped? ___________ Years

If the nodding stopped: does the patient now presents another form of epilepsy?
☐YES ☐NO
If YES: complete the following questions

Other forms of seizures

Has the participant had a seizure in the last 5 years?

☐ YES ☐ NO ☐ DON'T KNOW

What is the number of seizures since onset?

☐ One attack ☐ Two or more seizures

If only two seizures, were they more than 24h apart? ☐ YES ☐ NO ☐ DON'T KNOW

Aura / sensation (hearing, seeing, tasting, smelling, feeling) before seizures:

☐ YES ☐ NO ☐ DON'T KNOW

Episodes of loss of consciousness ☐ YES ☐ NO ☐ DON'T KNOW

Seizures with passing urine or stool on self and /or foaming at the mouth

☐ YES ☐ NO ☐ DON'T KNOW

Has he the participant had a seizure within the last 12 months?

☐ YES ☐ NO ☐ DON'T KNOW

IF YES, in which month has the last seizure been experienced?

MONTH: _ _ ☐ DON'T KNOW

What is the current frequency of the seizures?

☐ Yearly (if less than 1 per month)

☐ Monthly (if less than 4 per month)

☐ Weekly (if less than 7 per week)

☐ Daily (if more than 7 per week)

Specify number: _____________per ______________

How many seizures did you have LAST WEEK? ☐ Number____ ☐ None ☐ DON'T KNOW

Was the onset of seizures within the first year of life?

☐ YES ☐ NO ☐ DON'T KNOW

IF the onset of the seizures was within the first year of life:

☐ During the first 10 days of life
☐ More than 10 days to 6 month
☐ More than 6 month to 1 year

If the onset of seizures was after the age of one, at what age?

_____ years
☐ DON'T KNOW

Of what type are the most frequent seizures?

☐ Generalized seizures with loss of consciousness

☐ Atonic seizures (drop attacks)
☐ Absences
☐ Simple partial (focal) seizures (consciousness not lost)
☐ Complex partial (focal) seizures (decreased consciousness)
☐ Secondarily generalized partial seizures
☐ Others, specify: ______________________________

MEDICAL HISTORY

Family history of nodding disease ☐ YES ☐ NO ☐ DON'T KNOW
IF YES: Number of affected siblings with nodding syndrome _______

Family history of seizures ☐ YES ☐ NO ☐ DON'T KNOW
IF YES, specify who these are (tick all that apply)
☐Siblings (brother/sister); No. of affected siblings ______
☐Father ☐Mother ☐Grandparent(s)

Family history of mental illness ☐ YES ☐ NO ☐ DON'T KNOW

Questions for the mother of the participant
Did the pregnancy of the mother of the participant proceed normally?
☐ YES ☐ NO ☐ DON'T KNOW
If NO, specify: ____________________________________

Mode of delivery for the affected child:
☐ Spontaneous Vaginal Delivery
☐ Assisted Vaginal Delivery
☐ Caesarean section

Was the interviewed participant born at term (pregnancy had completed 9 months)?
☐ YES ☐ NO ☐ DON'T KNOW

Did the interviewed participant cry immediately?
☐ YES ☐ NO ☐ DON'T KNOW

What was the birth weight? ______________________GRAMS ☐ DON'T KNOW

Psychomotor Development during Childhood:

Prior to onset of seizures

Was the child growing normally prior to the onset of the seizures?
☐ Yes ☐ No ☐ DON'T KNOW
IF NO, at what age did the abnormal growing appear? ______ Years

Did the child learn to do things like other children of his/her age prior to the onset of the seizures? ☐ Yes ☐ No ☐ DON'T KNOW
IF NO, at what age did the learning difficulty start? ______ Year

Compared with other children of his/her age, did the child appear in any way mentally backward, dull or slow before the onset of the seizures?

☐ Yes ☐ No ☐ DON'T KNOW

IF YES, at what age did it start? ______ Years

Since the onset of seizures,

Compared with other children of his/her age, did the child learned to do things like other children?

☐ Normal ☐ Delayed ☐ Abnormal
☐ Others, specify: ________________________________

Compared with other children of his/her age, did the child appeared in any way mentally backward, dull or slow?

☐ Normal ☐ Delayed ☐ Abnormal
☐ Others, specify: ________________________________

Occurrence of severe disease in the past:

Has the interviewed participant suffered from severe measles before the onset of epileptic seizures?

☐ YES ☐ NO ☐ DON'T KNOW

Has the interviewed participant suffered from a severe form of malaria before the onset of epileptic seizures?

☐ YES ☐ NO ☐ DON'T KNOW

Has the interviewed participant suffered from encephalitis/meningitis before the onset of epileptic seizures?

☐ YES ☐ NO ☐ DON'T KNOW

Has the participant had a head injury with loss of consciousness before the onset of epileptic seizures?

☐ YES ☐ NO ☐ DON'T KNOW

Has the participant had a prolonged post-traumatic coma before the onset of epileptic seizures?

☐ YES ☐ NO ☐ DON'T KNOW

Has the patient presented febrile convulsions in the past? ☐ YES ☐ NO ☐ DON'T KNOW

Was the onset of epilepsy following another illness?

☐ YES ☐ NO ☐ DON'T KNOW

If YES, specify the illness:

____

Has the participant a history of excessive alcohol consumption?

☐ YES ☐ NO ☐ DON'T KNOW ☐ Not applicable*

Has the participant a history of drugs abuse?
☐ YES ☐ NO ☐ DON'T KNOW ☐ Not applicable*

GENERAL EXAMINATION

BODY WEIGHT (kg): _________ . ____kg

HEIGHT (cm): _______________cm

How is the general condition of the interviewed participant?
☐GOOD ☐ CORRECT ☐ POOR

Thoracic abnormalities ☐ YES ☐ NO

If yes specify ____

Facial abnormalities ☐ YES ☐ NO
If yes specify ____

Does the adolescent (> 16years old) /adult looks like a child? ☐ YES ☐ NO

If yes, external signs of sexual development conform to age:
☐ YES ☐ NO ☐ EXAMINATION DECLINED

If NO, specify: girls: ☐ breast not developed
girls and boys: ☐ no pubic hear

Ophthalmology ☐ NORMAL ☐ ABNORMAL VISION
☐ BLIND, BOTH EYES AFFECTED

Dermatology ☐ NORMAL ☐ ABNORMAL

Burn scars ☐ YES ☐ NO

Itching ☐ YES ☐ NO

Papular eruption ☐ YES ☐ NO

Depigmented lesions (leopard skin) ☐ YES ☐ NO

Suspected onchocerciasis nodules ☐ YES ☐ NO

NEUROLOGICAL EXAMINATION

Is the participant alert? ☐ YES ☐ NO

Fully oriented in place/time/person ☐ YES ☐ NO

Is the participant's cognitive development comparable with peers?
☐ YES ☐ NO

Normal vision ☐ YES ☐ NO

Normal hearing ☐ YES ☐ NO

Normal eye movements? ☐ YES ☐ NO

Generalised muscle wasting ☐ YES ☐ NO

Paresis ☐ YES ☐ NO
if YES specify____

Contractures ☐ YES ☐ NO

Is the participant walking normally? ☐ YES ☐ NO

IF NO, specify
☐ Ataxic (wide base) gait
☐ Waddling gait (like a duck)
☐ Spastic gait –with tip toe walking
☐ Hemiplegic – with one sided weakness
☐ Other, specify_____________________

Psychiatric symptoms

Does the participant has hallucinations, i.e. sees, hears, smells, feels or tastes things that don't exist?

☐ YES ☐ NO

Does the participant have delusions, i.e. strongly held false belief by participant despite superior evidence against belief? ☐ YES ☐ NO

Does the participant show aggressive episodes?
☐ YES ☐ NO

Have you felt very sad (irritable) for a period of more than two weeks? ☐ YES ☐ NO

Have you experienced loss of interest and pleasure in almost all activities for a period of more than two weeks? ☐ YES ☐ NO

In the past month, have you been having strong memories / dreams of something bad that happened to you or your loved one? ☐ YES ☐ NO

Does to participant suffer from another neuro-psychiatric / psychological problem?
☐ YES ☐ NO ☐ DON'T KNOW

IF YES for any of the questions about psychiatric symptoms, refer to neuro-psychiatrist:

Diagnosis neuro-psychiatrist (using the Acholi translated version of the MINI, a diagnostic tool for psychiatric disorders)

☐ Major Depression
☐ Post-traumatic Stress Syndrome
☐ Generalized Anxiety
☐ Pervasive Development Disorder

Physical / Functional Indices

Modified Rankin Scale: Please mark the most accurate description of the current functional state of the child, as observed during the evaluation

Score	Description
0	No symptoms at all
1	No significant disability despite symptoms; able to carry out all usual duties and activities
2	Slight disability; unable to carry out all previous activities, but able to look after own affairs without assistance
3	Moderate disability; requiring some help, but able to walk without assistance
4	Moderately severe disability; unable to walk without assistance and unable to attend to own bodily needs without assistance
5	Severe disability; bedridden, incontinent and requiring constant nursing care and attention
6	Dead
**SCORE (0-6): ________**	

CASE CLASSIFICATION

☐ Head Nodding Syndrome
☐ Head Nodding Syndrome plus
☐ Other form of Epilepsy
☐ Other diagnosis

If other diagnosis:

☐ Recurrent febrile convulsions
☐ Dizziness / syncope
☐ Paroxysmal vertigo
☐ Severe anaemia
☐ Mental retardation without epilepsy
☐ Psychiatric illness without epilepsy
☐ Classic migraine
☐ Other, specify_________

TREATMENT

What is or was the type of seizure medication taken by the participant?

☐ No treatment ☐ DON'T KNOW
☐ Traditional ☐ anti-epileptic drug
☐ Mixed

If anti-epileptic drug treatment: Which substance is taken by the participant (additionally you may check patient's treatment record)?

Phenytoin	☐ YES	☐ NO
Sodium valproate	☐ YES	☐ NO
Phenobarbital	☐ YES	☐ NO
Carbamazepin	☐ YES	☐ NO
Diazepam	☐ YES	☐ NO
Ethosuximide	☐ YES	☐ NO
Other anti-epileptic treatment	☐ YES	☐ NO

If YES, specify: ______________________________

Compliance: Is the participant taking the anti-epileptic drug treatment regularly?

☐ YES ☐ NO

If NO, why?
☐ Side effects
☐ (Temporary) non-availability of medication
☐ Lack of financial means to access medication
☐ DON'T KNOW
☐ Other, specify ____________________

If the person took anti-epileptic treatment what was the response to the treatment?

☐ No effect on the seizures
☐ Decrease of the frequency of seizures when the drug was taken
☐ No more seizures since drugs were taken
☐ DON'T KNOW

IVERMECTIN USE

Has the participant ever received ivermectin? ☐ YES ☐ NO ☐ DON'T KNOW

☐NOT APPLICABLE (according to exclusion criteria, as follows):
1) age <5 years at the moment of CDTi; 2) pregnancy; 3) Breast feeding < 7 days; 4) acute severe disease: ☐1 ☐2 ☐3 ☐4

IF YES: Has the participant taken ivermectin during the last CDTi in 2016?

☐ YES ☐ NO ☐ DON'T KNOW

How many times per year? ☐ ONCE ☐ TWICE ☐ DON'T KNOW

IF <u>NOT taken</u> in 2016, why? ☐ no distribution ☐ absent during the CDTi ☐ refused

☐ afraid of secondary effects
☐ pregnancy
☐ breastfeeding an infant younger than 7days
☐ because I was asked to NOT take it
☐ age <5 years at the time of CDTi

☐ severe acute disease at the time of CDTi
☐ other, specify ________________________________

IF <u>TAKEN</u> in 2016, why?
☐ It is recommended to be taken to prevent river blindness
☐ to decrease itching
☐ other, specify ________________________________

Was Ivermectin/Mectizan distributed in another way than orally?

☐ YES ☐ NO ☐ DON'T KNOW

Picture or video taken: ☐ YES ☐ NO

Action Taken by reporting officer: referred for treatment ☐ YES ☐ NO

If yes, where ___________________________

Signature of the interviewer _____________________________________
